# Nest defense in the face of cuckoldry: evolutionary rather than facultative adaptation to chronic paternity loss

**DOI:** 10.1186/s12862-019-1528-7

**Published:** 2019-11-04

**Authors:** Holger Zimmermann, Karoline Fritzsche, Jonathan M. Henshaw, Cyprian Katongo, Taylor Banda, Lawrence Makasa, Kristina M. Sefc, Aneesh P. H. Bose

**Affiliations:** 1 Institute of Biology, University of Graz, Universitätsplatz 2, 8010 Graz, Austria; 2 Department of Biological Sciences, University of Idaho, 875 Perimeter MS, Moscow, ID 3051 USA; 3 Department of Biological Sciences, University of Zambia, Great East Road Campus, P.O. Box 32379, Lusaka, Zambia; 4 Lake Tanganyika Research Unit, Department of Fisheries, Ministry of Fisheries and Livestock, P. O. Box 420055, Mpulungu, Zambia; 5 Present address: Department of Collective Behaviour, Max Planck Institute for Ornithology, Universitätsstraße 10, 78464 Konstanz, Germany

**Keywords:** Parental care, Multiple paternity, Cichlid, Paternal care adjustment, Parental investment, *Variabilichromis moorii*

## Abstract

**Background:**

Raising unrelated offspring is typically wasteful of parental resources and so individuals are expected to reduce or maintain low levels of parental effort when their parentage is low. This can involve facultative, flexible adjustments of parental care to cues of lost parentage in the current brood, stabilizing selection for a low level of paternal investment, or an evolutionary reduction in parental investment in response to chronically low parentage.

**Results:**

We studied parental care in *Variabilichromis moorii*, a socially monogamous, biparental cichlid fish, whose mating system is characterized by frequent cuckoldry and whose primary form of parental care is offspring defense. We combine field observations with genetic parentage analyses to show that while both parents defend their nest against intruding con- and hetero-specifics, males and females may do so for different reasons. Males in the study group (30 breeding pairs) sired 0–100% (median 83%) of the fry in their nests. Males defended less against immediate threats to the offspring, and more against threats to their territories, which are essential for the males’ future reproductive success. Males also showed no clear relationship between their share of defense and their paternity of the brood. Females, on the other hand, were related to nearly all the offspring under their care, and defended almost equally against all types of threats.

**Conclusion:**

Overall, males contributed less to defense than females and we suggest that this asymmetry is the result of an evolutionary response by males to chronically high paternity loss in this species. Although most males in the current study group achieved high parentage in their nests, the average paternity in *V. moorii*, sampled across multiple seasons, is only about 55%. We highlight the importance and complexity of studying nest defense as a form of parental care in systems where defense may serve not only to protect current offspring, but also to ensure future reproductive success by maintaining a territory.

## Background

Parental care has evolved in many species as a way for parents to increase their own reproductive success by enhancing the survival of their offspring. Because parental care demands time and energy it can incur fitness costs to the caregiver (i.e. parental investment [1]), and so parental care also presents an optimality problem [2]. A parent’s optimal investment into care should depend on the value of the current brood relative to the parent’s expected future reproductive success [3]. Brood value depends on numerous factors [4], including brood size [5–8], offspring survival prospects [9, 10], and parent-offspring relatedness [11]. Variable and uncertain parentage is common and taxonomically widespread [12, 13], and parents may use either direct cues (e.g. olfactory or visual) or indirect cues (e.g. social context) to assess their level of kinship to their broods [14]. A facultative reduction in parental investment is one way to mitigate the damage of compromised parentage, and such adjustments of parental care in response to cues of lost parentage have been described in some species (e.g. birds: reed buntings [15], blue tits [16], fishes: bluegill sunfish [17], pumpkinseed sunfish [18], plainfin midshipman fish [19], insects: taurus scarab [20]), but not in others [21–23]. Additionally, in species where paternity is often shared among multiple males, selection may act to reduce average paternal investment over evolutionary timescales [24–27].

A great deal of previous empirical research, largely conducted on birds, has investigated how parental care responds to reduced parentage. Typically, the rate of offspring provisioning – a highly demanding parental task [28] – is used to quantify parental care and act as a proxy for parental investment. Yet in many non-avian taxa, parental care rarely involves offspring provisioning, and in this respect fishes offer an interesting contrast to birds. With few exceptions, parental care in fishes does not involve provisioning young with food, but instead consists of fanning and cleaning eggs and defending young from predators [29, 30]. Parental care in fishes can still incur costs in terms of energetic expenditure (e.g. ref. [18, 31]), missed foraging opportunities (e.g. ref. [32]), exposure to predation risk (e.g. ref. [33]), and forfeiture or postponement of additional reproductive opportunities (e.g. ref. [34]), all of which are likely to affect future reproductive success. To date, most assays of parental defense behaviors in fish have involved presenting caregivers with a threat (i.e. a live or a model predator or competitor) and then monitoring the intensity of the caregivers’ response (e.g. ref. [18, 35–37]). While such methods can be useful for gauging a parent’s instantaneous motivation to defend, they lose ecological relevance because in the wild, parents rarely have a single threat to contend with and must partition their defense efforts against multiple simultaneous or consecutive intrusions. This highlights a research need to better understand defense as a component of parental care under natural conditions. Indeed, whereas behaviors such as offspring provisioning or fanning are largely depreciable forms of care determined by the needs and value of a particular brood, defense is typically non-depreciable and must additionally respond to the level of predation pressure in the environment. Consequently, optimal defense effort is challenging to predict based on brood value or parentage alone.

Many caregiving fishes are also highly territorial, with individuals competing for and defending territories that are subsequently used for rearing offspring in addition to attracting mates, foraging, and sheltering [29]. The behaviors involved in territory and brood defense can overlap, or even exactly coincide, because both involve driving territory intruders away. The study of how defense varies with brood value is thus complicated both by the dual purpose of defense behaviors and by temporal and spatial variation in intruder pressure. Furthermore, in species with biparental care, the amount of care provided by one parent may respond to the state and behavior of the other parent [38–40]. Sexual conflict between parents is expected to arise because each parent benefits when the other takes on a larger portion of the parental workload [41]. Sexual conflict can also be exacerbated when the value of the current offspring differs greatly between the parents. Such asymmetries in brood value occur, for example, when males are cuckolded [42], which is common in many fish mating systems [13]. Asymmetries in brood value could potentially lead to divergent evolutionary motivations for each parent to provide defense. For example, defense performed by females may serve primarily to protect young, while defense by males may be primarily to retain the territory and protect it from exploitation.

Here, we investigate how male brood defense relates to paternity in a sexually monomorphic, socially monogamous and biparental cichlid fish, *Variabilichromis moorii,* from Lake Tanganyika, East Africa. *V. moorii* is a herbivorous substrate-breeder [43, 44] and is very abundant (> 6 adults per 10m^2^) along rocky shorelines in southern Lake Tanganyika [45, 46]. Solitary individuals and mated pairs of the small (< 10 cm), dark-colored cichlid defend their territories against other members of the species-rich littoral fish community that compete with them for food and space. Additionally, non-territorial *V. moorii* cruise the rocky littoral, snatch up food and on occasion participate in the spawning of the mated pairs [47, 48]. Spawning females attach their clutches of up to > 100 eggs onto vertical rock surfaces. Free swimming larvae appear 4–5 days post spawning and are guarded by the parents for a period of up to 100 days, i.e. until grown to > 3 cm [44]. *V. moorii* broods have remarkable levels of extra-pair paternity, with brood-tending males siring an average of ~ 55% of the fry on their territories (range = 0–100%) and with 10 or more sires contributing to some broods [47, 49]. Females, on the other hand, have nearly absolute (~ 96%) maternity of the fry on their territories (small numbers of foreign fry, related to neither parent, are occasionally found within broods [47]). Despite this sex-asymmetry in parentage, both sexes defend the brood against intruding con- and heterospecifics [50–52]. In our study, we observed and scored defense behaviors of male and female parents on their territories under natural conditions, where intrusions by fry predators and territory competitors occur frequently [43, 50–52]. This allowed us to collect observational data on territory and brood defence from 31 breeding pairs without experimental manipulation of the nest environment. Paternity shares of the parental males were assessed based on microsatellite genotyping. We hypothesized that asymmetries between the parents in their defense labour (i.e. the number of defensive actions aimed towards intruders) would reflect asymmetries in brood value, which is greatly influenced by paternity in our study system. We also considered the possibility that the defense offered by each sex might differ depending on whether intruders represented threats primarily to the offspring (i.e. fry predators) or to the territory (i.e. risk of territory takeover or exploitation).

## Results

**Fig. 1:**
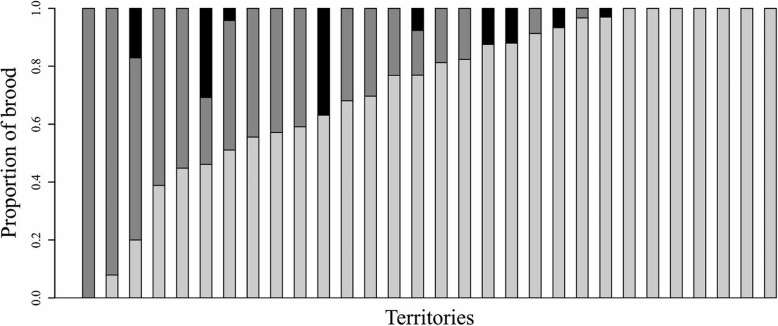
Brood parentage. For each genotyped brood, bars illustrate the proportions of within-pair offspring (light grey), offspring sired by extra-pair males (dark grey) and foreign fry (black), relative to total brood size. Note that paternity shares were calculated relative to maternal brood sizes, which do not include foreign fry

Maternal brood sizes ranged from 5 to 94 fry (median = 22 fry, interquartile range (IQR) = 20.5 fry, *n* = 31 territories); additionally, 9 of the nests contained 1–7 foreign fry. The total length of the fry ranged from 9 to 28 mm (mean ± s.d. 16.9 ± 5.0 mm, *n* = 31 territories). Brood sizes were not correlated with fry length, which we use here as a proxy for fry age (Spearman rank correlations; maternal brood size: *r*_*S*_ = − 0.05, *p* = 0.787; total brood size: *r*_*S*_ = 0.10, *p* = 0.592; n = 31 territories). Male total length (mean ± s.d. 8.3 ± 0.4 cm) was correlated with female total length (mean ± s.d. 8.4 ± 0.4 cm) within social pairs (Spearman rank correlation, *r*_*S*_ = 0.54, *p* = 0.0032, *n* = 31 territories), though neither sex was consistently larger than the other (paired Wilcoxon signed rank test, *V* = 134, *p* = 0.174, n = 31 territories). The within-pair size differences (female total length – male total length) ranged from − 0.7 cm to 0.8 cm (mean = 0.09 cm ± s.d. 0.37 cm, *n* = 28 pairs). Maternal brood sizes were positively correlated with the average total length of the parents (8.32 ± 0.34 cm; Spearman rank correlation, *r*_*S*_ = 0.42, *p* = 0.019, *n* = 31 territories). Paternity shares (relative to maternal brood size) of the territory-holding males ranged from 0% (in 1 territory) to 100% (in 12 territories), with a median of 82.8% (IQR = 42.4%, *n* = 30 territories; Fig. 1). The number of extra-pair sires per brood ranged from 0 to 8 (median = 1 extra-pair sire, IQR = 1.75 extra-pair sires, n = 30 territories).

### Intrusion pressure by brood predators versus territory competitors

**Fig. 2:**
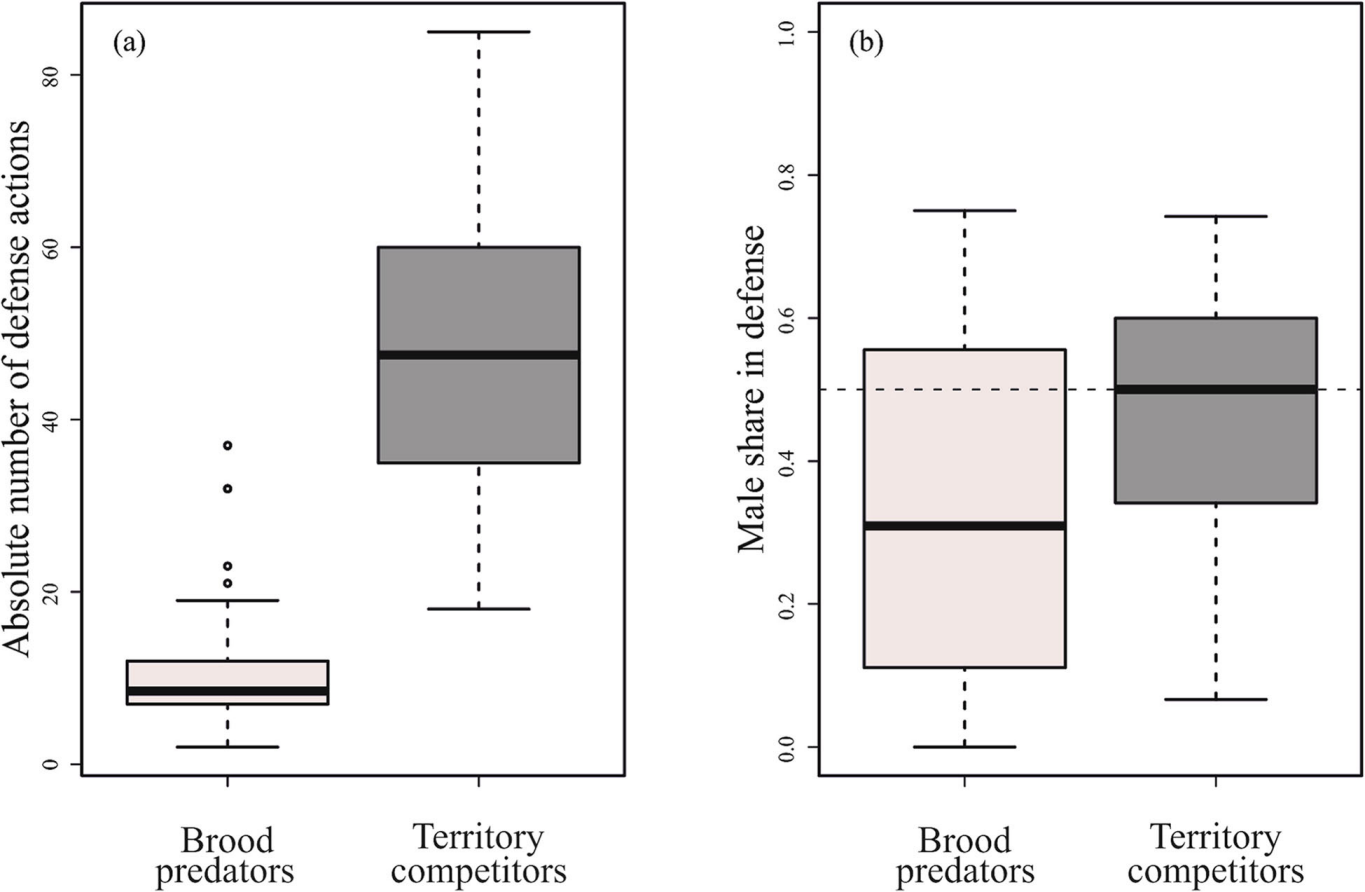
**a** Intrusion pressure from brood predators was lower than that from territory competitors. **b** Males contributed relatively less than their female partners to defense against brood predators than to defense against territory competitors. Horizontal dashed line indicates 0.5 (i.e. an egalitarian split of defense against intruders). Both (**a**) and (**b**) depict values per territory summed across the three observation periods

The observed pairs (n = 31) performed a median of 1.53 (range = 0.58–5.53, IQR 0.98) defense behaviors per minute. Since *V. moorii* consistently defended against unfamiliar, approaching fish, we used the total number of defense behaviors performed by the pair as a proxy for the intrusion pressure at that territory. Defense against brood predators (median of defense behaviors per min = 0.20, range = 0.07–2.13, IQR = 0.29) occurred less often than against territory competitors (median = 1.17, range = 0.40–4.00, IQR = 0.87, GLMM: est. = − 1.47, se = 0.088, z = − 16.68, *p* <  0.0001) (Fig. 2a). Intrusion pressure varied between the two intruder types depending on fry length (interaction, est. = 0.30, se = 0.092, z = 3.24, *p* = 0.0012) and average parental total length (interaction, est. = − 0.36, se = 0.11, z = − 3.45, *p* = 0.0006). Because of this, we present the results of two separate models, one for each intruder type, in Table 1. In brief, larger parents experienced more intrusion pressure from territory competitors. Intrusion pressure from territory competitors also decreased with increasing numbers of fry on a territory. Intrusion pressure from brood predators increased with increasing fry length.

**Table 1:** Effects of brood, parental, and territory variables on intrusion pressure by territory competitors and brood predators. Intrusion pressure was quantified as the number of defense behaviors performed by the breeding pair of *V. moorii* against each intruder type. Significant *p*-values (*p* < 0.05)  are in bold

	*Estimate*	*Std. Error*	*z*	*P*
Intrusion pressure from territory competitors				
(Intercept)	2.68	0.052	52.0	**< 0.0001**
Depth	0.085	0.055	1.54	0.12
Fry length	−0.002	0.054	− 0.04	0.97
Total brood size	−0.150	0.057	−2.63	**0.0085**
Average parent body size	40.291	0.061	4.83	**< 0.0001**
Intrusion pressure from brood predators				
(Intercept)	1.16	0.130	8.87	**< 0.0001**
Depth	−0.084	0.136	− 0.62	0.54
Fry length	0.331	0.132	2.52	**0.012**
Total brood size	0.037	0.119	0.32	0.75
Average parent body size	−0.095	0.130	− 0.74	0.46

### Relative contribution of males and females to defense against intruders

During the 45 min of cumulative observation time per territory, males and females were observed to engage in 0.07 (median, range = 0.00–0.47, IQR = 0.15) and 0.16 (median, range = 0.02–1.73, IQR = 0.22) defense behaviors per minute against brood predators respectively. Against territory competitors, on the other hand, males and females respectively engaged in 0.56 (median, range = 0.04–1.87, IQR = 0.51) and 0.60 (median, range = 0.18–2.53, IQR = 0.42) defense behavious per minute. When we pooled defense behaviors against brood predators with those against territory intruders, only the intercept term was significant, indicating a lower male share of defense relative to females (est. -0.347, se = 0.119, z = − 2.924, *p* = 0.0035; Additional file 1: Table S1). Paternity share, maternal brood size and the size difference between the caregivers had no detectable effect on male share of defense (Additional file 1: Table S1).

Male share of defense was significantly lower against brood predators (31% of the breeding pairs’ defense behaviors) than against territory competitors (47%; adding ‘intruder type’ as a predictor to above GLMM: est. -0.580, se = 0.171, z = − 3.390, *p* = 0.0007; Fig. 2b). We next investigated defense against each intruder type separately. Male share of defense against territory competitors was not significantly related to paternity, maternal brood size, or the size difference between the breeding partners (Table 2a), and there was no difference between male and female shares in defense against territory competitors (intercept term, Table 2a). Male share of defense against brood predators was similarly not significantly related to paternity, maternal brood size, or the size difference between the partners (Table 2b), but males did engage in significantly fewer defense behaviors against brood predators than their female partners (intercept term, Table 2b).

**Table 2:** Effects of paternity on male share of defense against territory competitors and brood predators. Maternal brood size and the size difference between females and males were included as additional factors that may affect male share of defense and/or male paternity share (see Methods). Significant intercept terms indicate non-egalitarian defense behaviors between males and females (note that parameter estimates are on the scale of the logit-link function). (a) Results for the model fit to intrusion pressure from territory competitors. (b) Results for the model fit to intrusion pressure from brood predators. Significant p-values (*p* < 0.05)  are in bold

	*Estimate*	*Std. Error*	*z*	*P*
(a) Intruder type: territory competitors				
(Intercept)	−0.175	0.125	−1.398	0.162
Paternity	0.439	0.487	0.901	0.368
Maternal brood size	0.001	0.124	0.010	0.992
Female – Male size difference	0.089	0.139	0.638	0.523
(b) Intruder type: brood predators				
(Intercept)	−1.004	0.268	−3.749	**0.0002**
Paternity	0.138	0.947	0.146	0.884
Maternal brood size	0.180	0.239	0.752	0.452
Female – Male size difference	−0.283	0.269	−1.051	0.293

## Discussion

In our study, females contributed significantly more than males to defense against brood predators (i.e. egg and fry predators as well as piscivorous species; henceforth called brood defense). In contrast, defense against territory competitors (mostly algae-eaters and zooplanktivores; henceforth called territory defense) was shared more equally between both parents (Fig. 2). Males participated more in territory defense than brood defense, but the relationships between either form of male defense and paternity or the other proxies of brood value were statistically unclear (sensu ref. [53]). We interpret these results as evolutionary responses to low paternity shares and different levels of motivation for brood and territory defense (see below). We also show that *V. moorii* parents face greater intrusion pressure from territory competitors than from brood predators. Intrusion pressure from brood predators increased with fry size, possibly because older fry are more mobile and conspicuous. Intrusion pressure from territory competitors increased with parent size, potentially because larger individuals hold larger and more valuable territories (but see ref. [50, 52]). Territory competitors also intruded more when brood sizes were small (Table 1), a pattern that could arise if algal cover in territories diminishes with the number of offspring grazing there, thus enhancing the foraging attractiveness (for herbivores) of territories with smaller broods. All of these possibilities require experimental verification in future studies.

### Males defend less than females against brood predators

In comparison to other fish species, the paternity of pair-bonded *V. moorii* males is especially low and variable [54– 59]. Pair-bonded, territory-holding male *V. moorii* achieve an average (± SD) of 57 ± 33% paternity in their broods (values collated from ref. [47] and unpublished data). Note, however, that the average paternity was higher in the subset of males considered in this study (72%, see Results section for details). In agreement with a previous study [52], male *V. moorii* contributed less than females to the total defense behaviors exibited by the pair. Here, we showed that this non-egalitarian share of defense is due to a deficit in male defense against brood predators specifically. The low participation of males in brood defense (31%) relative to females may thus represent an evolutionary response to chronically low average paternity in territory-holding *V. moorii*. Over evolutionary time, low average brood value can reduce paternal investment and/or maintain a low level of investment [22, 24, 26, 27, 60].

Sex-specific parental roles are common in biparental brood-caring fishes and have often been interpreted as division of labor associated with sexual size dimorphism; the larger individual, typically the male, emphasizes defense against intruders, while the smaller individual, typically the female, remains close to the brood and provides direct brood care such as fanning or cleaning (e.g. ref. [61–65]). *V. moorii*, however, are sexually monomorphic and social pair formation occurs between similarly-sized individuals (ref. [52]; our data). We could not find any statistically clear correlation between male share of defense and the body size differences seen within pairs. Though we cannot rule out whether other phenotypic traits that differ between the sexes (unrelated to body size) lead females to be more specialized in defense, both sexes appear similarly capable of performing defense behaviors. Overall, this supports the idea that differential brood defense between the sexes is linked to the asymmetry in average brood value. Data from recent studies suggest that in *V. moorii*, paired males likely do not have a higher expected future reproductive success than their female partners, which could have otherwise explained the lower degree of male parental expenditure. This is because males and females evidently engage in long-term pair-bonds and males apparently do not compensate for their lower parentage by reproducing outside of their pairs [47]. Differential brood defense also raises the question of how divergent evolutionary interests shape sex differences in parental care activities even in species without pronounced sexual dimorphism. We note, however, that sex biased parental care has even been observed in systems with lifelong genetic monogamy where brood value and reproductive opportunities do not differ between males and females (e.g. ref. [66]).

### Males and females defend to similar extents against territory competitors

While brood defense in *V. moorii* was female-biased, territory defense was shared in a more egalitarian manner, suggesting that both parents benefit similarly from protecting against territory competitors. However, unlike brood defense, territory defense is complicated by the fact that it may be motivated by multiple factors. On the one hand, territory defense may serve to protect against territory takeovers and exploitation. This may be a powerful motivator for defending, as the reproductive success of individuals is greatly enhanced by territory ownership [47]. On the other hand, territory defense still benefits current offspring because almost all the species we oberved, regardless of trophic specialization, consume fry opportunistically. Furthermore, offspring can suffer indirectly if territory competitors are permitted to take over the territory or graze the algae on which *V. moorii* feed. This highlights a challenging question: Why do parents evolve to defend against territory competitors – to protect the territory, the offspring, or both? Broadly, parental care is defined as a suite of parental traits (behavioral or non-behavioral) that has evolved and is currently maintained for the purpose of increasing offspring fitness [67]. It is therefore unclear how well territory defense fits the definition of parental care as such defense may enhance the probability of future reproductive success (by retaining the territory for future use) while also, perhaps incidentally, benefitting any current offspring. Future research focusing on teasing apart the relative importance of these factors will be important.

### Paternity does not relate to male brood defense

On average, males contributed less to brood defense than did females, which mirrors the males’ lower average relatedness to the broods (i.e. low average paternity but high maternity [47, 49]). However, when we looked at patterns of paternity and defence *within* the current study group, we did not find the expected positive correlation between an individual male’s brood paternity and his share of brood defense, which would indicate a facultative adjustment of paternal investment into the current brood.

It is important to consider how well *V. moorii* males satisfy the requirements under which a facultative adjustment of care to current paternity would be predicted [21, 22]. First, such adjustments are only predicted if males expect higher paternity in future broods. Overall, *V. moorii* broods show exceptionally variable paternity, both within and between seasons [47, 49]. Within-male variation in brood paternity can also be substantial: over 3 years of intensive sampling of a wild breeding population of *V. moorii* (5 field excursions in total), we obtained genetic paternity data from the broods of seven individual males that were recaptured on multiple occasions. The paternity of these males varied extensively across broods (average within-male standard deviation in brood paternity was 30.1%, see Additional file 2: Table S2). Thus, males faced with low current paternity can indeed expect higher success in future broods.

Second, reliable cues of parentage must be readily available for brood care to be adjusted in response to variable paternity. It is currently unclear whether *V. moorii* males have access to reliable cues, direct or indirect, of paternity loss. Males could foreseeably use the number of potential cuckolders intruding on a spawning event as a gauge of sperm competition intensity [17, 20]. Indeed, in our current study, sire number was strongly correlated with paternity of the paired male (Spearman correlation coefficient r = − 0.834). However, the number of rivals present during a spawning becomes an increasingly unreliable measure as the number of *unsuccessful* cuckolders in the group increases and becomes more variable [48]. Direct observations of *V. moorii* spawning events will be valuable for assessing the utility of this paternity cue. The use of direct offspring cues (e.g. olfactory kin recognition) can affect brood care decisions of cuckolded males [17] and has been demonstrated in several fish species, including cichlids [68–71]. However, it is currently unknown whether *V. moorii* males are sensitive to such direct cues. Future studies taking a more targetted approach at investigating the mechanisms of kin recognition in this species would be valuable.

Third, if care is to be adjusted in response to variable parentage, providing care must be costly in terms of reducing the parent’s residual reproductive value. While brood defense necessarily involves energetic expenditure [18, 32, 72], brood predators in this system pose little threat of predation or injury to the parents (i.e. negligible ‘risk investment’). Thus, it is possible that an evolutionary reduction in paternal investment in response to chronic paternity loss [24] may have rendered the costs of brood defense too low to warrant clear adjustments in care.

## Conclusions

High rates of cuckoldry and low average paternity over evolutionary time in *V. moorii* may have selected for a low baseline level of paternal relative to maternal investment. This was most evident for brood defense, in which males showed reduced participation in comparison to females. However, males engaged in similar amounts of territory defense as their female partners. Such results are in line with the idea that brood value is generally lower for males, but both partners benefit from long-term retention of their territory. We highlight the challenges of studing territory defense from the perspective of parental investment theory and emphasize the importance of uncovering the factors that motivate defense against territory competitors.

## Methods

### Field observations of brood defense and sampling

Field work took place in October and November 2015 at a study quadrat (area ≈ 1600m^2^ depth = 1.7–5.8 m) by Mutondwe Island, Zambia (8°42′29.4″S 31°07′18.0″E). The quadrat contained 85 territories with breeding *V. moorii*, from which we randomly selected 31 territories with free-swimming fry for behavioral observations (offspring spend most of the brood care period as free-swimming fry [44]). The two breeding adults from each territory were captured while on SCUBA, fin-clipped, sexed by examination of their genital papillae [52], and released. *V. moorii* adults are sexually monomorphic and so in order to distinguish between the male and female parent at each territory during subsequent observations, we clipped the caudal fins of the males and females differently (dorsal/ventral tip of the fin for males/females respectively). The fin clips were stored in 99.9% ethanol and later used for DNA-based parentage analyses (see below).

We measured the total length of the breeding fish to the nearest 0.1 cm. Two males and one female escaped after fin-clipping in the field before their length could be measured. However, given that *V. moorii* pair size-assortatively (ref. [43], current study) and that we were able to catch and measure their social partners, we linearly interpolated these missing body lengths (using the function ‘approx’, R package ‘stats’). This allowed us to preserve the sample size in all our analyses including parent sizes, although we note that omitting the territories with missing size data produced qualitatively similar results (Additional file 3: Table S3).

We started behavioral observations between 1 and 3 days after taking the fin clips. Fin-clipped individuals recover quickly, resuming parental care within 3 min after clipping (personal observations HZ, KF, JH, AB), and so the handling of the fish for clipping is unlikely to affect our observations, which occurred at least 24 h later. The same scuba diver (HZ) observed each territory for 15 min on each of three consecutive days (i.e. three 15-min observation sessions per territory). All observations took place before noon to avoid time-related differences in the behavior of the focal individuals as well as in the abundance of intruders. In two cases, the territory-holding male was absent for one of the three observation intervals. In one of these cases, the female was observed driving off her male partner repeatedly during the observation interval on the third day; in the other, the male was missing on the first day of observation. Since we were interested in the parental care decisions of the males, we avoided scoring behaviors that may not have been under the males’ control and omitted these 2 days from the analyses of parental care. During each observation interval, we tallied the defense behaviors of male and female territory-holders, which consisted of displays and overt aggression against fish intruding into the territory. We counted lateral displays (fish present their lateral side and erect fins to an intruder), frontal displays (fish face the intruder with lifted gill covers but not necessarily with erect fins) and overt aggression (attacks against intruders, which typically involved darting towards an intruder to chase it off). All intruders were identified to the species level, except for two species (*Telmatochromis dhonti* and *T. temporalis*) that could not be reliably distinguished.

After behavioral observations were completed, we collected all fry (882 fry from 31 territories), euthanized them in a bath of MS-222 (1 g / 1 L lake water), and preserved them in 99.9% ethanol for parentage analyses (see below). We estimated average fry length per brood by measuring the total length of four randomly chosen fry to the nearest mm. Here, fry length was used as a proxy for fry age. When broods contained fry of two or more different size classes, which occurs rarely due to foreign fry living on non-natal territories [47], we took measurements from four individuals in each size class, and then calculated a weighted average across all fry classes in the brood.

In our analyses, we distinguish between *total* brood size (all fry collected from a territory, including foreign fry in some territories [27 out of 882 fry in total were foreign]) and *maternal* brood size (fry attributable to the female territory-holder based on genetic parentage analyses). We also took fin clips for DNA extraction from an additional 158 adults captured within the quadrat (*n* = 219 total) to increase our sample size of adult *V. moorii* for estimating population allele frequencies. The fieldwork was carried out with the permission of the Fisheries Department of Zambia and under a study permit issued by the government of Zambia.

### Classification of intruders as brood predators and territory competitors

Breeding *V. moorii* defended against a wide variety of species, suggesting that defense may be motivated by both brood care and territoriality. In an attempt to distinguish between brood defense and territory-oriented defense, we classified the intruders into two groups (Table 3). *Brood predators* include well-documented egg and fry predators as well as piscivorous species [43, 51, 73]. The remaining species, many of which are algae-eaters or zooplanktivores, have been observed to hold territories similar to those of *V. moorii* and to occasionally take over territories from *V. moorii* (personal observation KF, HZ). These species were classified as *territory competitors*. Although most species will opportunistically consume eggs and fry, the species grouped together as brood predators are more specialised in doing so than any of the species within the territory competitor group. The observer (HZ) did not notice any attempts made by territory competitors to eat the defended fry, whereas they did observe this from brood predators, though none of the observed predation attempts were successful. None of the observed intruders pose a threat to adult *V. moorii*.

**Table 3:** Classification of species into territory competitors and brood predators. Total number of defense actions against each species during the observation of the focal territories is given in parentheses

Territory competitors	Brood predators
*Variabilichromis moorii* (657)	*Telmatochromis vittatus* (177)
*Telmatochromis temporalis & T. dhonti* (219)	*Neolamprologus fasciatus* (145)
*Eretmodus cyanostictus* (172)	*Lamprologus callipterus* (17)
*Neolamprologus modestus* (104)	*Ctenochromis horei* (15)
*Julidochromis ornatus* (84)	*Lepidiolamprologus elongatus* (12)
*Neolamprologus caudopunctatus* (50)	*Mastacembelus sp.* (11)
*Neolamprologus savoryi* (45)	*Altolamprologus compressiceps* (5)
*Lobochilotes labiatus* (13)	*Lepidiolamprologus attenuatus* (1)
*Lamprichthys tanganicanus* (6)	
*Ophthalmotilapia ventralis* (45)	
*Neolamprologus pulcher* (31)	
*Xenotilapia spilopterus* (28)	
*Neolamprologus tetracanthus* (27)	
*Tropheus moorii* (22)	
*Petrochromis famula* (11)	
*Aulonocranus dewindti* (7)	
*Interochromis loocki* (7)	
*Neolamprologus sexfasciatus* (6)	
*Neolamprologus mustax* (3)	
*Neolamprologus prochilus* (2)	
*Petrochromis polyodon* (2)	
*Simochromis diagramma* (2)	
*Haplotaxodon microlepis* (1)	

### Genetic parentage analysis

DNA extraction from fin clips of adult *V. moorii* followed an ammonium acetate precipitation protocol [74]. Genetic paternity could only be determined for 30 territories, because one breeding male could not be captured for fin-clipping. We followed a standard Chelex protocol [75] for extracting DNA from fry tissue (*n* = 882). After centrifugation for 5 min at 4000*g, tubes were stored at − 20 °C until PCRs were performed.

All samples were genotyped at 14 microsatellite loci in 2 multiplex reactions as described in Bose et al. [47]. The expected heterozygosity ranged from 0.700 to 0.946 (mean ± sd, 0.879 ± 0.077) and no deviation from Hardy-Weinberg equilibrium could be detected (for details see ref. [47]). We estimated population allele frequencies from the sample of 219 adult *V. moorii*, and parentage analyses were carried out for each brood separately with the help of COLONY (v 2.0.6.1, [76]). We estimated paternity share (percentage of brood sired by the brood-tending males), the absolute number of fry sired by the brood-tending male, and the number of extra-pair sires. Paternity share was estimated as the number of within-pair fry divided by the maternal brood size. We carefully checked the COLONY output for cases in which extra-pair fry were proposed on the basis of only one to three allele mismatches with the brood-tending males. Most of these cases could be resolved as genotyping errors by re-scoring electropherograms or repeating the PCR. In the few remaining cases, mismatches at one or two loci were considered to be due to mutations or unrecognized genotyping errors, whereas mismatches at more than two loci were assumed to indicate a different sire than the brood-tending male. We also identified cases in which COLONY presumed that there were two separate extra-pair males, each having sired a single fry, despite the two fry sharing the same mother. We saw no compelling reason to reject the more parsimonious assumption of one shared father in these cases, and corrected the estimated number of extra-pair sires accordingly.

### Statistical analyses

We used R v. 3.4.4 (R Development Core Team) for all statistical analyses. For the statistical models that follow, all continuous predictor variables as well as brood sizes (count variables ranging from 5 to 94 fry) were mean-centered and scaled by dividing by their standard deviations prior to analyses. Proportion variables (i.e. paternity) were mean-centered.

Since each unfamiliar (i.e. non-neighboring) fish that approached the *V. moorii* territory elicited a single defense response by one of the two breeding individuals, we used total defense (i.e. the sum of male and female defense actions) as a proxy for the intrusion pressure on the territory. We first tested whether intrusion pressure from brood predators differed from that of territory competitors and whether these pressures varied with properties of the broods or territories. To do this, we fit generalized linear mixed-effects models (GLMM, R package lme4 [77]) with poisson error distributions. We included total defense behaviors performed by the breeding pair per 15-min observation period versus each intruder type (count) as our response variable. We included intruder type (categorical variable: brood predator vs. territory competitor), total brood size, average fry length, territory depth, and average total length of the parental individuals as predictor variables. To account for non-independence due to the temporal grouping of observations, we also fit ‘territory ID’ nested within the ‘date of the territory’s first observation’ as a random effect. We also fit an observation-level random intercept to account for overdispersion [78]. We included the number of fry, as well as the sizes of the fry and parents, as predictor variables because of the possibility that larger individuals or groups are more conspicuous to intruders. We included territory depth in our model because predator density has previously been suggested to increase with depth [52]. Next, we focused on each intruder type separately and fit two GLMMs, as just described, for territory competitors and for brood predators independently.

Next, we tested whether males varied their share of defense in relation to brood value. We first fit a GLMM with a binomial error distribution, in which we ignored intruder type by pooling our data between brood predators and territory intruders. We included the proportion of defense behaviors, observed within each 15-min period, that was performed by the male (proportion based on count data) as our response variable (“male share of defense”). We included paternity share, maternal brood size, and the size difference between the caregivers (female total length minus male total length) as predictor variables. Since we were interested in asymmetries between the parents, we calculated paternity share relative to maternal brood size (as adopted young do not directly contribute to either parent’s fitness). We included ‘day of observation’ (i.e. day 1, 2 or 3) and ‘territory ID’ as random intercepts and also included an observation-level random intercept to account for overdispersion [78]. To test whether males contributed differently to defense against brood predators than they did against territory competitors, we added ‘intruder type’ as a predictor to this GLMM. Finally, we fit two separate GLMMs, one for each intruder type, to address differences in male behavior against brood predators and territory competitors. Note that the intercept terms in these GLMMs test whether defense is shared equally between males and females.

## Supplementary information

**Additional file 1: Table S1.** Effects of paternity on male share of defense, when pooling across intruder types. Maternal brood size and the size difference between females and males were included as additional factors that may affect male share of defense and/or male paternity share (see main text). The significant intercept term indicates non-egalitarian defense behaviors between males and females (note that parameter estimates are on the scale of the logit-link function).

**Additional file 2: Table S2.** Variation in within-male paternity shares. Paternity of breeding males recaptured over three years of sampling was determined using nine microsatellite markers (Pmv17, Pzeb3, TmoM11, UNH2075, Hchi59, Hchi94, Ppun9, Ppun20, Ppun21; see main text), and is given in percent of maternal brood size.

**Additional file 3: Table S3.** Results of statistical models for intrusion pressure and male share in defense after excluding nests with missing parental size values. (A) and (B) show results corresponding to those in Tables 1 and 2 of the main text, respectively.

**Additional file 4: Table S4.** Nest descriptions and observed defense behaviors.

## Data Availability

The data used in this paper are available in Additional file 4: Table S4.

## Abbreviations

GLMM: Generalized linear mixed model
IQR: Interquartile range
PCR: Polymerase chain reaction
