# Supplementary material for: Nest defense in the face of cuckoldry: evolutionary rather than facultative adaptation to chronic paternity loss
Source: BMC Evol Biol. 2019 Nov 4;19:200. doi: 10.1186/s12862-019-1528-7 (PMC6829816; doi:10.1186/s12862-019-1528-7)
Supplement: Supplementary file 1 — Additional file 1: Table S1. Effects of paternity on male share of defense, when pooling across intruder types. Maternal brood size and the size difference between females and males were included as additional factors that may affect male share of defense and/or male paternity share (see main text). The significant intercept term indicates non-egalitarian defense behaviors between males and females (note that parameter estimates are on the scale of the logit-link function). [file 12862_2019_1528_MOESM1_ESM.docx]

**Table S1:** Effects of paternity on male share of defense, when pooling across intruder types . Maternal brood size and the size difference between females and males were included as additional factors that may affect male share of defense and/or male paternity share (see main text). The significant intercept term indicates non-egalitarian defense behaviors between males and females (note that parameter estimates are on the scale of the logit-link function).

|  | | *Estimate* | *Std. Error* | *z* | *P* |
| --- | --- | --- | --- | --- | --- |
|  | (Intercept) | -0.291 | 0.122 | -2.39 | **0.0168** |
|  | Female-Male size difference | 0.033 | 0.135 | 0.25 | 0.81 |
|  | Paternity | 0.452 | 0.471 | 0.96 | 0.34 |
|  | Maternal brood size | 0.019 | 0.121 | 0.16 | 0.87 |
