# Supplementary material for: Nest defense in the face of cuckoldry: evolutionary rather than facultative adaptation to chronic paternity loss
Source: BMC Evol Biol. 2019 Nov 4;19:200. doi: 10.1186/s12862-019-1528-7 (PMC6829816; doi:10.1186/s12862-019-1528-7)
Supplement: Supplementary file 2 — Additional file 2: Table S2. Variation in within-male paternity shares. Paternity of breeding males recaptured over three years of sampling was determined using nine microsatellite markers (Pmv17, Pzeb3, TmoM11, UNH2075, Hchi59, Hchi94, Ppun9, Ppun20, Ppun21; see main text), and is given in percent of maternal brood size. [file 12862_2019_1528_MOESM2_ESM.docx]

**Table S2:** Variation in within-male paternity shares. Paternity of breeding males recaptured over three years of sampling was determined using nine microsatellite markers (Pmv17, Pzeb3, TmoM11, UNH2075, Hchi59, Hchi94, Ppun9, Ppun20, Ppun21; see main text), and is given in percent of maternal brood size.

|  | *October 2015* | *April 2016* | *October 2017* | *April 2018* | *September 2018* |
| --- | --- | --- | --- | --- | --- |
| male 1 |  | 100 | 69 |  |  |
| male 2 |  | 100 |  | 48 |  |
| male 3 | 100 | 8 | 45 |  |  |
| male 4 |  |  |  | 15 | 100 |
| male 5 |  |  | 50 |  | 24 |
| male 6 |  |  | 100 |  | 69 |
| male 7 |  |  | 36 | 44 |  |
