# Supplementary material for: Nest defense in the face of cuckoldry: evolutionary rather than facultative adaptation to chronic paternity loss
Source: BMC Evol Biol. 2019 Nov 4;19:200. doi: 10.1186/s12862-019-1528-7 (PMC6829816; doi:10.1186/s12862-019-1528-7)
Supplement: Supplementary file 3 — Additional file 3: Table S3. Results of statistical models for intrusion pressure and male share in defense after excluding nests with missing parental size values. (A) and (B) show results corresponding to those in Tables 1 and 2 of the main text, respectively. [file 12862_2019_1528_MOESM3_ESM.docx]

**Table S3:** Results of statistical models for intrusion pressure and male share in defense after excluding nests with missing parental size values. (A) and (B) show results corresponding to those in Table 2 and Table 3 of the main text, respectively.

|  | | *Estimate* | *Std. Error* | *z* | *P* |
| --- | --- | --- | --- | --- | --- |
| (A)  Intrusion pressure from **territory competitors** | | | | | |
|  | (Intercept) | 2.699 | 0.053 | 50.46 | **< 0.0001** |
|  | Depth | 0.091 | 0.056 | 1.637 | 0.102 |
|  | Fry length | 0.003 | 0.056 | 0.061 | 0.951 |
|  | Total brood size | -0.142 | 0.057 | -2.483 | **0.013** |
|  | Average parent body size | 0.291 | 0.061 | 4.773 | **< 0.0001** |
|  | |  |  |  |  |
| Intrusion pressure from **brood predators** | | | | | |
|  | (Intercept) | 1.164 | 0.137 | 8.478 | **< 0.0001** |
|  | Depth | -0.080 | 0.134 | -0.592 | 0.554 |
|  | Fry length | 0.347 | 0.139 | 2.503 | **0.012** |
|  | Total brood size | 0.051 | 0.122 | 0.422 | 0.673 |
|  | Average parent body size | -0.101 | 0.131 | -0.768 | 0.443 |
|  |  |  |  |  |  |
| (B)  Male share in total defense versus **territory competitors** | | | | | |
|  | (Intercept) | -0.229 | 0.128 | -1.789 | 0.074 |
|  | Paternity | 0.291 | 0.487 | 0.598 | 0.550 |
|  | Maternal brood size | -0.014 | 0.123 | -0.113 | 0.910 |
|  | Female – Male size difference | 0.066 | 0.138 | 0.479 | 0.632 |
|  | |  |  |  |  |
| Male share in total defense versus **brood predators** | | | | | |
|  | (Intercept) | -1.033 | 0.298 | -3.473 | **0.0005** |
|  | Paternity | 0.216 | 1.034 | 0.209 | 0.835 |
|  | Maternal brood size | 0.184 | 0.257 | 0.713 | 0.476 |
|  | Female – Male size difference | -0.282 | 0.291 | -0.970 | 0.332 |
