# Supplementary material for: Nest defense in the face of cuckoldry: evolutionary rather than facultative adaptation to chronic paternity loss
Source: BMC Evol Biol. 2019 Nov 4;19:200. doi: 10.1186/s12862-019-1528-7 (PMC6829816; doi:10.1186/s12862-019-1528-7)
Supplement: Supplementary file 4 [file 12862_2019_1528_MOESM4_ESM.docx]

## Sheet: male_defense

Table S4: Nest descriptions and observed defense behaviors.  
nest ID	depth of nest location (m)	fry length (mm)	sires per nest	extra-pair sires per nest	offspring sired by nestholding male	maternal brood size	total brood size	paternity share of nestholding male	total length, female (cm)	total length, male (cm)	male defense behaviors - brood predators	female defense behaviors - brood predators	male defense behaviors - territory competitors	female defense behaviors - territory competitors
nest1	3.5	19	2	1	26	44	44	0.590909091	9	8.7	0	2	23	31
nest2	3.8	16	2	1	64	94	94	0.680851064	8.5	8.3	7	5	22	38
nest3	4.7	19	1	0	13	13	13	1	8	8.1	0	11	22	13
nest4	3.1	22.8	2	1	6	9	15	0.666666667	8	8.1	9	23	10	26
nest5	3	22	2	1	23	33	38	0.696969697	9.1	8.8	3	11	37	37
nest6	4.2	17	2	1	29	30	31	0.966666667	8.2	7.9	3	4	23	13
nest7	3.6	18	1	0	33	33	41	1	8.5		2	5	23	8
nest8	4.8	12	2	1	14	17	17	0.823529412	8.5	8.3	0	7	42	39
nest9	4.6	13	2	1	10	12	13	0.833333333	7.9	7.5	8	11	14	27
nest10	2.8	11	1	0	5	5	6	1	8.2	8.1	4	2	25	33
nest11	3	22	1	0	11	11	13	1	8.2	7.7	2	6	35	19
nest12	1.8	12	2	1	32	34	36	NA	8.5	NA	5	13	18	11
nest13	2.2	23.3	7	6	7	29	54	0.24137931	9.1	8.9	7	5	41	28
nest14	2.1	19.9	6	5	24	45	47	0.533333333	8.7	8.5	18	19	30	20
nest15	4.3	13	2	1	21	23	23	0.913043478	8.2	8	6	4	31	34
nest16	2.4	9	4	3	10	18	18	0.555555556	8.3	9	5	4	16	62
nest17	1.7	14	1	1	0	13	13	0	8	8.6	0	8	14	14
nest18	2	28	1	0	14	14	15	1	8.2	8.6	14	7	34	25
nest19	2.4	24	1	0	7	7	9	1	8.5	8	2	4	34	29
nest20	3.1	15	1	0	12	12	12	1	8	8.1	3	1	18	16
nest21	2.9	12	3	2	12	21	21	0.571428571	8	7.2	0	8	2	16
nest22	2.9	10	5	4	7	18	18	0.388888889	8.1	8.6	0	5	2	28
nest23	2.2	18	3	2	13	29	29	0.448275862	8.9	8.5	8	15	25	27
nest24	2.1	15.6	3	2	13	16	18	0.8125	8.7	8.6	1	2	53	32
nest25	2.6	15	9	8	3	38	41	0.078947368	8.1	8.5	9	3	15	22
nest26	1.8	13	2	1	43	56	62	0.767857143	8.1	8.2	1	7	15	10
nest27	3	12.7	1	0	38	38	39	1	8.1	8	2	3	15	14
nest28	4	10	1	0	23	23	23	1	NA	8	2	6	28	17
nest29	3.6	21	1	0	12	12	19	1	8.6	8.2	6	26	17	34
nest30	4.2	22	1	0	63	63	78	1	8.7	8.9	1	8	10	32
nest31	3.9	23.2	1	0	22	22	28	1	8.6	8	0	9	8	27
